# Diabetes Screening and Prevention in a High-Risk, Medically Isolated Border Community

**DOI:** 10.3389/fpubh.2017.00135

**Published:** 2017-06-12

**Authors:** Ann V. Millard, Margaret A. Graham, Nelda Mier, Jesus Moralez, Maria Perez-Patron, Brian Wickwire, Marlynn L. May, Marcia G. Ory

**Affiliations:** ^1^Texas A&M School of Public Health, McAllen, TX, United States; ^2^Department of Sociology & Anthropology, The University of Texas Rio Grande Valley, Edinburg, TX, United States; ^3^The University of Texas School of Public Health, Brownsville, TX, United States; ^4^Texas A&M School of Public Health, College Station, TX, United States; ^5^Nuestra Clinica del Valle, San Juan, TX, United States

**Keywords:** diabetes, prediabetes, diabetes prevention, diabetes education, health disparity, Hispanic, Mexican American, Latino

## Abstract

**Introduction:**

A project in a Texas border community setting, Prevention Organized against Diabetes and Dialysis with Education and Resources (POD^2^ER), offered diabetes prevention information, screening, and medical referrals. The setting was a large, longstanding flea market that functions as a shopping mall for low-income people. The priority population included medically underserved urban and rural Mexican Americans. Components of the program addressed those with diabetes, prediabetes, and accompanying relatives and friends.

**Background:**

People living in the Lower Rio Grande Valley (LRGV) face challenges of high rates of type 2 diabetes, lack of knowledge about prevention, and inadequate access to medical care. Recent statistics from actual community-wide screenings indicate a high diabetes prevalence, 30.7% among adults in the LRGV compared with 12.3% nationwide.

**Methods:**

A diverse team composed of public health faculty, students, a physician, a community health worker, and community volunteers conceived and developed the project with a focus on cultural and economic congruence and a user-friendly atmosphere. The program provided screening for prediabetes and diabetes with a hemoglobin A1c test. Screening was offered to those who were at least 25 years of age and not pregnant. When results indicated diabetes, a test for kidney damage was offered (urinary albumin-to-creatinine ratio). A medical appointment at a community clinic within a week was provided to those who tested positive for diabetes and lacked a medical home. Health education modules addressed all family members.

**Discussion:**

The project was successful in recruiting 2,332 high-risk people in 26 months in a community setting, providing clinic referrals to those without a doctor, introducing them to treatment, and providing diabetes prevention information to all project participants. Implications for research and practice are highlighted.

**Conclusion:**

This study shows that a regular access point in a place frequented by large numbers of medically marginalized people in a program designed to eliminate cultural and economic barriers can succeed in providing a hard-to-reach community with diabetes prevention services. Aspects of this program can serve as a model for other service provision for similar populations and settings.

## Introduction

People living in the border region of Texas closest to the Gulf of Mexico, known as the Lower Rio Grande Valley (LRGV), face challenges of high rates of type 2 diabetes, lack of knowledge about prevention, and inadequate access to medical care. A recent, scientifically rigorous study found that the prevalence of diabetes in the region is 30.7% among adults (≥18 years of age) compared with 12.3% nationwide (≥20 years of age) (1, 2). This article focuses on a project located in Hidalgo County, with a population of 849,843 (3) including many medically uninsured (38% of those <65 years in 2013) (4). Diabetes hospitalization rates are among the highest in Texas (20.0 per 10,000, State Health Services Region 11; compared with 17.1 statewide) (5). The diabetes mortality rate in Hidalgo County (23.8, age-adjusted rate per 100,000 population) is higher than that in the state (21.6) (6). Currently, diabetes is the leading cause of death in Mexico (7), the country of origin of many current residents in the region and their ancestors. Reversing the diabetes epidemic is crucial for communities on both sides of the border.

Nuestra Clinica del Valle, the local federally qualified health center, has a patient load of approximately 30,000 people, one-third of whom have diabetes, which tends to be diagnosed late in the course of the disease. Medically uninsured patients are forced into a pattern of visiting the hospital emergency department in diabetic crisis, resulting in annual costs of $284,655 per patient for emergency dialysis compared with $76,906 for Medicare-reimbursed, maintenance dialysis, which is more frequent, safer, and healthier (8, 9). These costs are from a study in Houston, TX, USA, and they are consistent with costs in the region of this study according to interview data. Delayed diagnosis is devastating emotionally and throughout the economy in the budgets of households, businesses, counties, Medicaid, and uncompensated care.

Many people lack information about the causes of type 2 diabetes and, especially, the crucial risk reduction processes of improving patterns of eating and physical activity. Local people of all income levels worry about the devastation caused by diabetes and hope for a medical cure for the disease, which they currently see as a death sentence, and indeed, diabetes-caused amputation and blindness are encountered routinely in local communities. Thus, scientific research and general public concern point to diabetes as a significant problem for health and family well-being, and preventive information is not reaching those who most need it.

Given these concerns, the local community sought assistance from the McAllen Campus of the Texas A&M School of Public Health. To engage in diabetes prevention, public health students and faculty and healthcare providers formed a team to create a project in a location frequented by people with limited incomes and tenuous access to medical care. As it developed, the project took on a complex set of activities, as reflected by the project name, POD^2^ER, Prevention Organized against Diabetes and Dialysis with Education and Resources in English and Prevención Organizada contra la Diabetes y Diálisis con Educación y Recursos in Spanish. *Poder* means power in Spanish, reflecting the project focus on empowering participants through eliminating barriers to medical care and preventive information.

The priority population addressed by POD^2^ER was urban and rural people who have tenuous access to medical services, limited incomes, and speak mostly Spanish. The barriers addressed by the project were temporal, economic, cultural, geographic, architectural, and informational. The project site was a longstanding *pulga* (“flea market”) that resembles the kind of market found by the plaza at the center of a Mexican town, but it is much larger, 58.5 acres. The market has long rows of indoor and outdoor booths, a large hall for musical performances and dancing, and shops selling most of the things one would find at a mall, from shoes to cleaning supplies, haircuts, full-sized clothes washers, new and used clothes, toys, pets, snacks and meals, and in addition, fresh fruits and vegetables. The *pulga* is open on Saturdays and Sundays, and visitors usually come with their families and spend hours browsing, eating, shopping, and enjoying music, live and pre-recorded. Because it draws a large, low-income clientele from a large urban and rural geographic catchment area, the *pulga* provided an ideal location for POD^2^ER. At our booth, visitors did not want to take much time because they had come to shop, mall walk, and socialize with their family members. They had limited incomes, and nearly all were Mexican American Spanish speakers. Regarding architecture, the booth had to be upgraded to house a lab and bathroom used for collecting urine samples to test for diabetic nephropathy. The project had to adapt information from evidence-based materials aimed at readers and listeners with greater health and scientific literacy.

The purpose of this article is to describe the rationale for the POD^2^ER project within its local context, highlight its development through a community-participatory framework and delineate its essential ingredients, overview intervention reach and outcomes, and discuss how POD^2^ER serves as a best-practice model that evolved over time, including implications for both research and practice.

## Background and Rationale

The initial question of the project was whether the community of interest would engage with the project and follow through on medical referrals. The project design combined findings of previous studies and some of the principles of community-based participatory research in public health. Innovations of the project were the choice of the delivery site, the elevation of a community health worker to take a major role in project design, staff training, and running the day-to-day operations of the project, and the development of culturally tailored evidence-based information and curriculum for prevention education.

Research shows that interventions delivered to Hispanic border residents by community health workers result in significant improvements in glycemic control (10). The work of community clinics and community centers in the LRGV routinely involves community health workers, called *promotores de salud* (*promotoras* for those who are women). They provide a crucial element of translational services between Spanish and English and between complex medical information and explanations comprehensible to people with low health literacy. A promotora played a key role in the development of this intervention.

Community-based diabetes screenings can be effective in assessing diabetes risk in rural individuals (11–13). Many of the participants in our project live in *colonias*, which are defined in Texas as rural, unincorporated settlements with weak infrastructure. (“Colonia” has a different meaning in Mexico and other parts of the U.S.) Hidalgo County is large and mostly rural, with over 1,000 colonias lying outside a string of small cities paralleling the Rio Grande. Colonias dot the landscape in rural areas; most are settlements made up of parcels of agricultural land sold off by aging farmers or their heirs with minimal investment in infrastructure. A colonia tends to be located in a food desert or food swamp (i.e., an area with food that is accessible but unhealthy) (14). The residents customarily shop at the *pulga*, a market with multiple vendors modeled on town-center markets in Mexico that draws customers from surrounding rural and urban communities. Our project team was thus inspired to locate the project in the largest *pulga* in the area to recruit the medically underserved in the region.

The American Diabetes Association (ADA) recommends screening in the community only when tied directly to medical treatment for those with positive results for diabetes. Screening programs can contribute importantly in prevention and cost effectiveness (15–18). Our project was formulated with the inspiration and collaboration of a volunteer physician from Nuestra Clinica del Valle, and he facilitated appointments for referrals.

Diabetes prevention programs that are culturally tailored are effective in reducing the risk of diabetes among Hispanics (19–21). Our project included many culturally congruent elements to make it accessible to local limited-income Mexican Americans. We combined these elements to reduce barriers to screening and diabetes prevention information to raise the likelihood that participants and their families would act to improve their health as early as possible in the course of the development of diabetes.

A study by Vissman et al. (22) found that immigrant Hispanics obtain prescription medicine and medical advice at informal non-medical locations, including *tiendas* (small grocery stores) and from family and social networks. At the site of our project, a number of vendors offered herbal and other remedies, including plants, to control diabetes. The market context thus was culturally appropriate for our project.

## Methods: Essential Elements of the Intervention

Prevention Organized against Diabetes and Dialysis with Education and Resources incorporated the following elements supported by the literature: a promotora, community-based diabetes screenings, a culturally tailored program, and a project site in an environment where the priority population felt comfortable.

**Figure 1:**
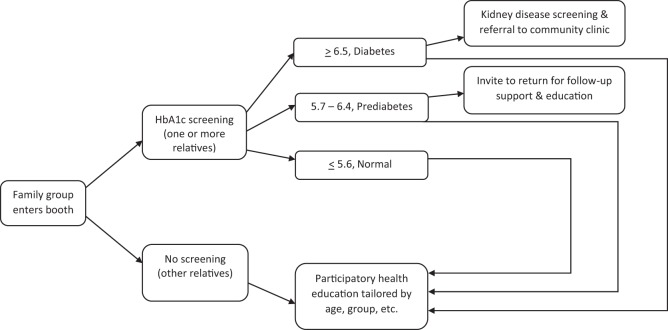
Decision tree: screenings, health education, and referral.

The project had three main elements (see Figure 1 for details):

(1) Health education and increased awareness. All visitors were provided with information about healthy eating, physical activity, and risk factors for diabetes. *Pláticas* (diadactic, participatory discussions) were provided with food models, recipes, and other information in an interactive format for individuals and groups often involving entire families. Young children were invited to use crayons to draw vegetables on paper plates, which were then exhibited in the booth. In summary, this program was able to provide a small amount of targeted education to those who tested positive for diabetes or prediabetes and their family members at the point of care.
(2) Screening for diabetes and prediabetes. POD^2^ER offered screening to those who were ≥25 years old and who were not pregnant. The screening tested hemoglobin A1c levels, and those with indications of diabetes were offered screening for kidney disease with a urinary albumin-to-creatinine ratio (ACR). All participants were provided with papers showing their test results, weight, and height. Those with indications of diabetes were referred for medical care.
(3) Follow-up. Referrals were provided to a community clinic for those who lacked a doctor. The community clinic has the sliding fee scale required for federally qualified health centers for the benefit of low-income patients; referrals from POD^2^ER were given free laboratory tests for a free first visit as well as a free second visit. Clinic staff followed up with phone calls to those who missed their appointments. At the POD^2^ER booth, those with prediabetes were invited to return in 3–6 months to see whether they had improved their A1c levels and to receive further health education. All participants and visitors were invited to attend platicas (discussions) at the booth to learn about diabetes prevention.

Prevention Organized against Diabetes and Dialysis with Education and Resources included a number of innovative features including project team composition of a promotora, public health graduate students and faculty, and an M.D., facilitating cultural tailoring of materials and procedures. The initial idea for the project came from a class of one coauthor (May), where another coauthor (Wickwire) gave a presentation inspiring four of the students (including Moralez, coauthor) to develop the project for their Master of Public Health practicum requirements with support from another coauthor (Millard) (23, 24).

Prevention Organized against Diabetes and Dialysis with Education and Resources extended the role of the promotora in recruitment and team management in day-to-day operations. The promotora had previous experience in community-based diabetes prevention and research projects in colonias. She was also a trained medical assistant with experience with the testing machines. In this project, she directed the Master of Public Health students, faculty, physician, and other program staff in recruiting participants, registering them and obtaining informed consent, and managing their flow through the service site. As a medical assistant with considerable clinical experience, she also oversaw the maintenance of and supplies for the testing equipment, calibration of machines, and many other details. The promotora possessed an unusual combination of experience and training; however, it would be possible to expand this project to other sites by separating the roles of the service site manager and a medical assistant overseeing calibration and testing.

The multiple components of the project made implementation complex, involving staff training on testing hemoglobin A1c and ACR in addition to social and educational components. We were fortunate to have access to the Siemens DCA Vantage Analyzer machine, which is Clinical Laboratory Improvement Amendments (CLIA)-waived and more accurate and consistent than earlier HbA1c machines and hand-held A1c testing devices. (CLIA-waived means that the test has been found to have a low risk of erroneous results under regulations related to the CLIA of 1988.) Data from referrals showed that the Siemens results are consistent with those of a more complex, highly accurate machine, the Tosoh G-8 machine in the local community clinic.

With community interest and support, the project grew significantly over a year’s time; POD^2^ER was able to pay to upgrade four adjoining market booths to create two large rooms that were enclosed and heat-controlled and included a bathroom for obtaining urine specimens. The design was developed by the project team. The architecture included windows to the outside and between the two rooms, providing visibility while guarding privacy. Space for children included a large window into the laboratory to allow them to see their parents and *vice-versa*. The colors were cheerful; furniture was folding chairs and tables; posters and handouts were displayed on the walls.

The ongoing regular weekly schedule, Sundays from 9:00 a.m. to 1:00 p.m., made the project a reliable access point. The regular schedule allowed effective word-of-mouth recruitment, and it allowed people with prediabetes to return for follow-up in 3–6 months. Recruitment involved some innovative and useful elements. An initial point of contact outside the booth was a board with bottled commercial drinks attached showing their sugar contents. The board stimulated conversation with passersby, often leading to their entry into the project booth. The exhibit thus provided both brief health education and participation.

An effective initial phrase for inviting participation was, “Free diabetes test” (“Gratis; prueba de diabetes”). Family and friends often urged someone to enter. If people were passing by and seemed to be thinking about participating, our staff would encourage them to enter on the spur of the moment. Once the person was inside, the process of informed consent would be implemented. When people said they had already eaten and thus could not have a blood sugar test, the staff would respond with a brief explanation that the hemoglobin A1c test does not require fasting and reflects the blood sugar level for the past 3 months. If someone declined to participate initially, a flier was provided with an invitation to return when convenient. This step was important in generating participation. If someone responded that they already knew they had diabetes, we would invite them to come in for recipes and other information.

Information was developed in English and Spanish for participants who had limited health literacy. Although bilingual materials were available from the Centers for Disease Prevention and Control (CDC) and the ADA, they were written at too complex a level for most of our participants. Our guiding principles were to provide education in brief, highly targeted formats, and to design handouts with attractive pictures and few words. For example, our flier advertising the project included health education images.

Measuring height and weight was a matter that had to be handled in private. Although the cultural preference is to be overweight with a stocky body shape, being obese is regarded as shameful. The promotora thus developed a routine in which a participant would be measured and weighed and then shown how to trace those results on a body mass index chart delineating obesity, overweight, and other categories. Many people were surprised and dismayed to learn that they were obese, and this information was best arrived at by themselves rather than from project staff.

The project provided an ADA version of MyPlate that showed a glass of water, not milk, because a majority of Mexican Americans are lactose intolerant and in local culture, drinking milk is unusual among adults. POD^2^ER was designed therefore to include dairy products as optional. The version of MyPlate offered to participants also included an image with half the plate in vegetables. MyPlate versions often show about one quarter of the plate in fruit; however, this project has not emphasized fruit because it is already very popular among local people. Vegetables are not favored as much, particularly among men.

The project developed recipes that follow local traditions, for example, beans, cabbage soup, and tacos, adjusted to include more vegetables and no simple carbohydrates, dairy food, or meat. Project staff collected information about how participants talk about diabetes and prevention, and we used that information to craft health education. For example, someone whose test indicated prediabetes commented that he was now on “the tightrope” (“la cuerda floja”), and we used that image to communicate about the nature of prediabetes.

## Results

The project screened 2,332 people from November 2014 through December 2016. Men often tend to avoid preventive primary care, but this project was able to screen a relatively high percentage of males, 46% (see Table 1). The high participation rate of men results from the setting and the urging of their accompanying family members to take advantage of the opportunity for screening. Ninety-eight percent of participants were Hispanic. On average, the participants had 9.1 years of education. Completing *secundaria*, which is equivalent to ninth grade, is accepted as an adequate education in much of Mexico, and many of the participants were Mexican immigrants who had lived in the U.S. for a long time.

**Table 1:** Sociodemographic characteristics and insurance status of Prevention Organized against Diabetes and Dialysis with Education and Resources participants (*n* = 2,332).

Characteristics	
Age, mean (SD), years	47.5 (11.1)
Gender (%)	46% male, 54% female
Educational attainment in years of school completed, mean (SD), years	9.1 (4.1)
**Ethnicity (%)**	
Hispanic	98%
**Insurance status (%)**	
Has medical coverage	29%

### Prediabetes and Diabetes

Of those screened, 25.3% tested positive for diabetes and 28.5% for prediabetes (see Table 2). Those whose tests indicated normal blood sugar were only 46.2%. Of those with diabetic results, 19.3% had an HbA1c level of 6.5–6.9, indicating controlled diabetes. Many participants (66%) already had had an earlier blood sugar test, many by using a glucometer of a friend or relative. Those who were not in medical treatment were making use of others’ glucometers but without understanding the test results.

**Table 2:** Health characteristics of Prevention Organized against Diabetes and Dialysis with Education and Resources (POD^2^ER) participants (*n* = 2,332).

Body mass index	
Normal (<25)	12%
Overweight (25–29)	35%
Obese (≥30)	53%
Screening results, HbA1c	
Mean (SD)	6.4 (1.9)
Normal (≤5.6)	46.2%
Prediabetic (5.7–6.4)	28.5%
Diabetic (≥6.5)	25.3%
Previously had blood sugar screened	66%
HbA1c at POD^2^ER indicated diabetes (≥6.5) and previously diagnosed with diabetes by an MD	65%

Among those whose tests indicated diabetes, many (65%) already had a doctor’s diagnosis of diabetes. Of the participants with diabetic results, 61% agreed to a urine test. The results showed that 27% already had moderately or severely increased ACRs, indicating kidney damage. Most would have been able to use medical treatment to minimize kidney damage or at least stabilize it; however, damage was advanced among 2% of those tested for ACR, meaning that they were very likely to be in dialysis within 5 years at substantial annual cost, as much as $284,655 per person for emergency dialysis.

### Nutrition Education

Regarding the nutrition education component, several observations were striking. Adults and children, men and women alike would state, “I don’t eat vegetables” and “I don’t drink water.” These pronouncements were made with an assertive tone, with a sense of pride or perhaps, in the spirit of small acts of resistance in everyday life (25, 26). Their alternative to water, unfortunately, was soft drinks. Among participants, 43% of men and 31% of women reported that they drank soft drinks daily during the previous week; about one-third drank them three or more times a day. Those asserting they did not eat vegetables in the previous week included 15% of men and 6% of women; 13% of men and 8% of women reported eating no fruit during that time.

A second promotora joined the team in the last 6 months and provided basic nutrition education using food models. Nearly all participants were surprised at how small the recommended portion sizes were and at the large amounts of sugar in soft drinks, the problems of refined carbohydrates, the value of whole-grain foods, and the benefits from eating a large quantity of vegetables. Those who did understand these concepts were the few participants who already had longstanding medical treatment for diabetes with thorough health education.

## Discussion

This study examines an innovative program in a setting frequented by limited-income community members in the Texas–Mexico border region. POD^2^ER was successful in providing screening for diabetes and prediabetes, referrals, and health education and awareness to communities with tenuous access to medical care. A second successful aspect of the program was that most of those with referrals attended their medical appointments according to follow-up data from the community clinic. Information on preventing diabetes was given to all participants and to any passersby who stopped to look at learning materials outside the booth. For every person screened, the project reached about four more people as measured through counts of visitors to the booth who were not screened.

The fact that many participants in screening had been diagnosed by a doctor was surprising to us. Many of them, however, reported that they felt fine and did not see the point of getting medical treatment. There is suspicion locally that doctors overtreat and overtest patients to make more money, with some support in the literature (27, 28). It is also important to recognize that physicians receive almost no training in nutrition, leaving them poorly equipped to advise patients on healthy eating to prevent chronic disease (29). On the other hand, among those participants who began medical treatment at the community clinic, some returned to the booth to thank us with reports of feeling much healthier, almost in a state of disbelief that they could feel so much better in a week or two.

Regarding healthy eating, the participants tended to have poor patterns of intake. It is commonly thought that meat is an acceptable meal and that vegetables are not necessary. Those who did eat vegetables often described it as a childhood habit instilled by their mothers. In colonias, communities tend to believe that their tap water is contaminated, an idea widespread in Mexico as well, giving people a health reason for drinking soft drinks. Some diabetics who were on medication would assert proudly that they had not changed their eating patterns and said they were doing well, potentially as a multifaceted response to the disease and advice about it (30, 31). For diabetics, more effective nutrition education is a crucial requirement. A major shift in eating by individuals and families toward less obesogenic and diabetogenic patterns is quite unusual in local communities.

The initial project priority was to identify people with diabetes and get them into medical treatment. The project also included upstream efforts, that is, components in primary public health prevention to reduce the incidence of diabetes on the population level. Specifically, people with prediabetes needed effective health education to prevent their developing diabetes, and their family members needed the same because they form the immediate social environment of those desperately needing to change their patterns of eating and physical activity. Success in these upstream endeavors would reduce the number of people with diabetes. For these reasons, the project moved to a larger space allowing greater emphasis on health education of all visitors. It is increasingly obvious, furthermore, that the entire community needs information on healthy eating, physical activity, and diabetes prevention; the information should not be limited to health education in a clinical setting.

### Implications for Practice

The basic screening program in this project can serve people of all income levels and it is scalable. The greatest impact would be among the medically underserved. A promotora who was knowledgeable of local cultural views and behaviors and comfortable in working with researchers and physicians was key in shaping our project to reach people with limited incomes and directing the project team at the service site.

At the beginning of the project, team members consulted materials from the CDC and the ADA. The project team members were surprised that we could not identify materials that would communicate well to our priority community. Therefore, we developed evidence-based materials that were effective at the flea market.

The project eliminated barriers to access to diabetes prevention on several levels. First, and very importantly, the project provided free screening and initial laboratory tests and appointments at a community clinic. Many participants lacked health insurance and the rest would have faced co-pay requirements at most other clinics. Whereas the community clinic will serve people at no cost if they cannot pay for services, patients face barriers of documentation requirements that were absent in this project. Second, the engagement of family members on-site was important to generating social support and understanding for those being screened. Third, the open and friendly demeanor of the project staff helped to put people at ease. In many cases, the staff member who interviewed the participant also weighed, measured, and tested that person, with the result that rapport was established before the discussion of improvements in patterns of eating and physical activity. Finally, the layout of the booth with windows in the outside passage and between the reception and education room and the laboratory room helped to make the project self-explanatory and user-friendly.

The epidemiologic context of the project made it suitable for a screening, referral, and education project in a public setting. The local adult diabetes prevalence of 30.7% created an urgent need for widespread screening coupled with a medical appointment within the week for those whose tests indicated diabetes. The project’s high rate of detection of positive cases, 25.3% of those screened, made implementation efficient. A project of this nature would not be worthwhile in a population with a low prevalence of diabetes because the cost per case detected would be much higher, making other methods of identifying those at high risk of diabetes more efficient. It is also important to note that although patterns of eating and physical activity can change on the individual level, the region of this study also needs improved access to healthy food and better infrastructure for those traveling by foot, bicycle, and public transportation. Thwarting the diabetes epidemic cannot be achieved one person at a time in the absence of environmental change.

Most families in the LRGV have a relative at risk of or living with diabetes, and many believe the disease is just a part of normal aging among Hispanics (32). Our results show that despite widespread familiarity with diabetes in the region, many are unaware of their own status and many who have already been diagnosed do not follow a treatment plan until their symptoms become extreme and interfere with activities of daily living.

On the other hand, prediabetes is not a familiar concept among local people, and some local doctors say that there is no such condition (Millard, field notes 2002–2017). Project participants were often greatly relieved when their tests indicated prediabetes, and they generally saw no reason to take preventive action. Nonetheless, the project staff emphasized the importance of immediate prevention through improved nutrition and physical activity. These ideas are difficult to comprehend, however, in a thoroughgoing obesogenic and diabetogenic environment.

### Implications for Research

Although successful in many aspects, our experience with POD^2^ER provides important avenues for further research and intervention design. One project weakness is that the encounter at the project site tends to be extremely brief, allowing as little as 10 min for transmitting information about how to improve eating and physical activity. Different formats for platicas are important to explore, especially for prediabetics and their family members.

Second, and related to the obstacle of the briefness of the encounter, health education information also needs to be evidence-based, appropriate culturally and economically, and provided in a participatory format. Many diabetes self-management education curricula are based on simplified overviews of nutrition expertise, but perhaps this approach is inappropriate for communities with limited health literacy. Is there a simpler, evidence-based way to provide them with the basic information that they need?

Third, although the project is successful in beginning treatment of those with diabetic test results, keeping them in treatment is difficult. How a community clinic can best provide ongoing diabetes care—i.e., care for those with chronic conditions—is an important question. If the project team and other researchers can devise effective, efficient ways to address diabetes and prediabetes, the findings may apply to many low-income Hispanic communities across the country.

## Conclusion

Prevention Organized against Diabetes and Dialysis with Education and Resources is an innovative, community-driven, and community-sited project aimed at diabetes control and prevention in a medically isolated population on the Texas–Mexico border. A team of local people with a wide range of health knowledge and socioeconomic status designed and staffed the project. The project was successful in recruitment and referral. The project developed health education and awareness materials and techniques for diabetics, prediabetics, and family members. The conclusion is that a screening and referral project located in a community setting can successfully introduce medically isolated Hispanics in a border population to effective medical treatment for diabetes.

## Ethics Statement

This study was carried out in accordance with the rules of the Human Research Protection Program, Institutional Review Board, Texas A&M University. Written informed consent was granted by most subjects in accordance with the Declaration of Helsinki. Data from other people were included as a pre-existing, de-identified data set on participants who had received information about potential inclusion in statistical analyses and who had voluntarily participated. The entire protocol was approved by the Human Research Protection Program.

## Author Contributions

All authors have made substantial, direct, and intellectual contribution to the work and approved it for publication.

## Conflict of Interest Statement

The authors declare that the research was conducted in the absence of any commercial or financial relationships that could be construed as a potential conflict of interest.
